# An Overview of Vasculogenic Mimicry in Breast Cancer

**DOI:** 10.3389/fonc.2020.00220

**Published:** 2020-02-27

**Authors:** Marco A. Andonegui-Elguera, Yair Alfaro-Mora, Rodrigo Cáceres-Gutiérrez, Claudia Haydee Sarai Caro-Sánchez, Luis A. Herrera, José Díaz-Chávez

**Affiliations:** ^1^Unidad de Investigación Biomédica en Cáncer, Instituto Nacional de Cancerología-Instituto de Investigaciones Biomédicas, UNAM, Mexico City, Mexico; ^2^Departamento de Genética y Biología Molecular, Centro de Investigación y de Estudios Avanzados del IPN, Mexico City, Mexico; ^3^Departamento de Patología, Instituto Nacional de Cancerología (INCAN), Mexico City, Mexico; ^4^Dirección General, Instituto Nacional de Medicina Genómica, Mexico City, Mexico

**Keywords:** vasculogenic mimicry, breast cancer, angiogenesis, cancer stem cell, epithelial-mesenchymal transition, triple negative breast cancer

## Abstract

Vasculogenic mimicry (VM) is the formation of vascular channels lacking endothelial cells. These channels are lined by tumor cells with cancer stem cell features, positive for periodic acid-Schiff, and negative for CD31 staining. The term VM was introduced by Maniotis et al. (1), who reported this phenomenon in highly aggressive uveal melanomas; since then, VM has been associated with poor prognosis, tumor aggressiveness, metastasis, and drug resistance in several tumors, including breast cancer. It is proposed that VM and angiogenesis (the *de novo* formation of blood vessels from the established vasculature by endothelial cells, which is observed in several tumors) rely on some common mechanisms. Furthermore, it is also suggested that VM could constitute a means to circumvent anti-angiogenic treatment in cancer. Therefore, it is important to determinant the factors that dictate the onset of VM. In this review, we describe the current understanding of VM formation in breast cancer, including specific signaling pathways, and cancer stem cells. In addition, we discuss the clinical significance of VM in prognosis and new opportunities of VM as a target for breast cancer therapy.

## Background

Breast cancer is the most prevalent malignant tumor in women worldwide. Approximately 2.1 million cases were diagnosed in 2018, and it is the leading cause of cancer death in women (2). According to the WHO, breast cancer is classified histologically into invasive carcinoma, and other specific types, such as invasive lobular carcinoma, metaplastic carcinoma, carcinoma with medullary factor, among others (3, 4). However, chemotherapy of breast cancer is determined by another tumor classification. Up to 70% of invasive breast tumors show estrogen receptor alpha (ERalpha) or progesterone receptor (PR) expression. This group of patients is treated with ER-alpha inhibitors or aromatase inhibitors alone or in combination with standard chemotherapy (taxanes plus anthracyclines). About 20% of the patients have amplification or overexpression of the *ERBB2* gene (HER2/neu). For these patients, treatment includes the use of antibodies directed against the *ERBB2-*encoded protein, which is a receptor of the EGFR family, and small molecules that inhibit the tyrosine kinase activity of the receptor. Finally, there is a group of tumors in which none of these markers is detected; these tumors are called triple-negative breast cancer (TNBC). They are a heterogeneous group of tumors with unfavorable prognosis, in which standard chemotherapy is used (5, 6). Recently, new therapies for breast cancer have been approved. For example, the use of talazoparib or olaparib (poly (ADP-ribose) polymerase inhibitors (PARP) enzymes) in patients with mutations in BRCA1 and BRCA2 (7). CDK4 and CDK6 kinase inhibitors have been approved as a therapy for patients with estrogen receptor-positive and HER2-negative tumors (8). Patients with the same type of tumors, bearing mutations in the PIK3CA gene have been approved for PI3K kinase inhibitors (9). In the case of TNBCs, a high percentage have been shown to exhibit expression of PD-L1, a PD-1 ligand that inactivates the immune response. In this group, atezolizumab (a humanized anti-PD-L1 antibody) has been approved for use in combination with nab-paclitaxel (10, 11).

On the other hand, while a tumor is growing, hypoxic zones are formed due to the lack of blood vessels. Tumor vessel formation can occur through angiogenesis, i.e., the development of new blood vessels from pre-existing ones. When stimulated by the tumor, endothelial cells from normal vessels begin to migrate and proliferate, forming new vessels inside the tumor. Tumor angiogenesis is regulated by the VEGF (Vascular Endothelial Growth Factor) and the transcription factor HIF1alpha (Inducible Hypoxia Factor 1alpha). Discovery of some factors that regulate angiogenesis has led to the development of specific drugs that block this process, such as antibodies against VEGF (Bevacizumab) or molecules like sunitinib or sorafenib, which inhibit crucial kinases in angiogenesis (12, 13). In breast cancer, angiogenesis is considered a poor prognostic factor for survival (14). However, anti-angiogenic therapies in breast cancer have not demonstrated benefit in overall survival as adjuvant treatment or in metastatic disease (14, 15).

## Vasculogenic Mimicry and Related Signal Pathways

**Figure 1:**
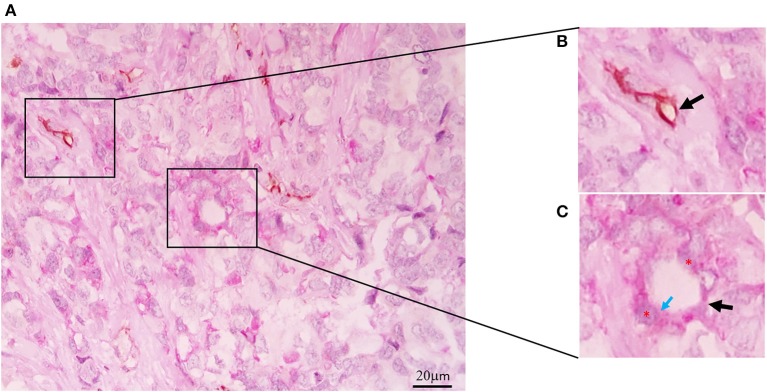
Vasculogenic mimicry in Triple Negative Breast Cancer. **(A)** CD31-PAS Double-staining (magnification 40x). **(B)** CD31-positive endothelial vessel (black arrow). **(C)** Tubular-type vasculogenic mimicry (VM) channel (black arrow), PAS-positive cuboidal tumor cells (red asterisks), PAS reaction in the luminal surface (blue arrow).

In 1999, Maniotis et al. described the formation of tumor vessels lacking endothelial cells in uveal melanoma. These vessels were positively stained with periodic acid-Schiff (PAS), and they did not possess endothelial cell markers such as Factor VII-related antigen or CD31. They had a characteristic pattern and erythrocytes inside. In highly invasive cell lines grown in matrigel, structures similar to tumor vessels with cells positive for PAS and negative for CD31 were observed. Besides, these structures allowed the perfusion of a dye, showing that they were functional vessels. This phenomenon was termed vasculogenesis mimicry (VM) (1). Subsequently, VM was reported in other tumors, such as breast, ovary, prostate, and lung, among others (16–18). Positive PAS staining without CD31 detection (PAS+CD31–) is the most widely used marker for defining the presence of VM (Figure 1). The presence of erythrocytes in the vessels and their perfusion capacity suggest that they can irrigate tumors to avoid hypoxia and to transport nutrients. In addition, the presence of VM has been associated with the appearance of metastasis (19).

**Figure 2:**
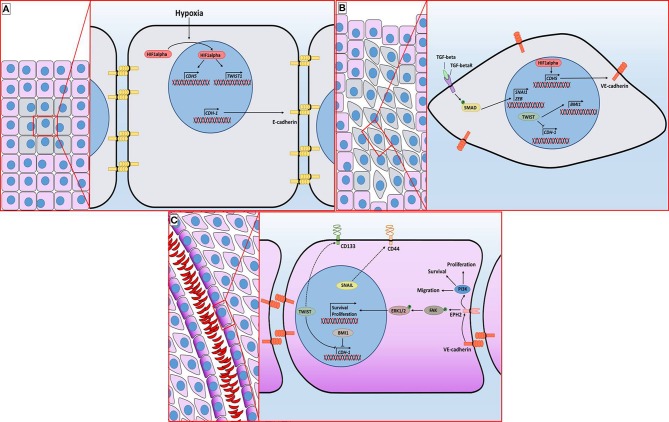
Molecular mechanisms associated with vasculogenic mimicry in breast cancer. **(A)** The incipient malignant tumor retains its epithelial architecture through adherens junctions mediated by E-Cadherin but has an innermost hypoxic core (gray cells). Hypoxia promotes the stabilization of HIF1alpha, which is followed by its translocation to the nucleus, granting access to its target genes. **(B)** TGFβ signaling and hypoxia-induced *TWIST* expression promote the epithelial-mesenchymal transition. E-Cadherin loss provokes a distortion of the epithelial architecture. **(C)** The sequence of molecular events initiated by hypoxia ultimately leads to the acquisition of cellular features associated with VM vessel formation, including the presence of EPH2 and CD44 in the plasma membrane. The purple gradient of the cells lining the lumen of the VM vessel is intended to represent PAS staining. Dotted line indicates an indirect interaction.

Since the discovery of VM, several factors regulating the formation of these vessels have been described. Like in the case of angiogenesis, hypoxia promotes VM. In cell lines derived from esophageal carcinoma, it was observed that inhibition of HIF1alpha inhibits the formation of VM and decreases the levels of proteins involved in the creation of these vessels, such as VE-cadherin, EPHA2 (ephrin A2) and Laminin 5gamma2 (20, 21). VE-cadherin is a relevant protein in VM. Under normal conditions, VE-cadherin is located in the plasma membrane of endothelial cells where it regulates intercellular unions. However, it has been observed that it is overexpressed in cells capable of performing VM. VE-cadherin is positively regulated by VEGF and by HIF1alpha (Figure 2A). VE-cadherin directs the location of EPHA2 to the intercellular junctions between cells that form the characteristic tubes of VM. EPHA2 is a kinase that activates two essential pathways in VM: PI3K (phosphoinositide 3-kinase) and ERK1/2 (extracellular signal-regulated kinase 1/2) (through FAK kinase) which are associated with survival, proliferation, and migration (Figure 2C). PI3K also allows the activation of MMP14 (matrix metalloproteinase-14) which in turn activates MMP-2. This metalloproteinase cuts laminin 5gamma2, producing gamma2' and gamma2x fragments, which promote cell migration. Inhibition of the factors involved in VM signaling prevents the formation of vessels (16, 22, 23).

On the other hand, some microRNAs (miRNAs) are related to the regulation of vascular mimicry. MiRNAs are non-coding 19-to 24-base RNAs that control gene expression by binding to mRNAs, usually in the 3′untranslated region (3′-UTR). MiRNAs can decrease transcription or prevent translation. In cancer, different microRNAs have been found to modify the regulation of oncogenes and tumor suppressor genes (24). MicroRNAs also regulate VM by interacting with specific genes; for example, miR-141 controls the expression of *EPHA2*. A decrease in miR-141 expression has been observed in high-grade gliomas. Besides, in glioma-derived cell lines, a decrease in miR-141 is associated with an increase in EPHA2 and an increase in VM (25).

## Factors Involved in VM in Breast Cancer

The presence of VM in breast cancer has been associated with poor prognosis in several clinical parameters (Table 1). Overexpression of factors regulating VM in breast tumors, such as HIF1alpha, VE-cadherin, and EPHA2 has also been reported (33–36). In a mouse breast cancer model, inhibition of angiogenesis promoted VM by expression of VE-Cadherin and other VM regulators in triple-negative tumors (37). In the MDA-MB-231 cell line (derived from a triple-negative tumor) which can form a pattern of tubular structures in matrigel, low expression of miR-204 was observed, while overexpression of miR-204 decreased the VM. This study also showed that PI3K-alpha and c-SRC are targets of miR-204. Therefore, it was proposed that miR-204 regulates critical pathways in VM, such as PI3K, MAPK, and SRC (38). Another factor associated with VM in breast cancer is osteopontin, a phosphoprotein related to tumor progression in different types of cancer. In a spheroid model of cell lines derived from breast tumors, an increase in osteopontin expression was observed in cells that formed vessels in matrigel. The expression of osteopontin was associated with a decrease in hsa-mir-299-5p, which targets osteopontin (39). Besides, in a study that analyzed 200 breast cancer patient samples, an association of the presence of Osteopontin and VM was observed (27). On the other hand, in the MDA-MB-231 cell line, overexpression of WT-1 isoforms (Wilm's tumor 1) promoted VM, by increasing the expression of EPHA2 and VE-cadherin (40). The enzyme DDAH1 (dimethylarginine dimethylaminohydrolase-1) has also been associated with the formation of VM: inhibition of DDAH1 in MDA-MB-231 cells prevents the formation of VM. Interestingly, miR-193b decreases the levels of DDAH1 and, therefore, inhibits the formation of VM (41, 42). Other miRNAs regulate VM in breast cancer. In endothelial cells, cisplatin treatment was shown to promote the production of IL-6, which, through the STAT3 signal transducer, promotes cisplatin resistance and vessel formation by VM in MDA-MB-231 cells. The miR-125a targets *IL-6* and *STAT3*. Decreased levels of miR-125a in endothelial cells were associated with increased production of IL-6 which promotes vessel formation by VM in breast cancer cells (43). On the other hand, the non-coding long RNA TP73-AS1 was shown to decrease the levels of miR-490-3p, which negatively regulates the *TWIST1* gene. TWIST participates in the epithelial-mesenchymal transition and promotes the formation of VM. Therefore, the expression of TP73-AS1 stimulates the formation of VM through the overexpression of *TWIST1* (44).

**Table 1:** Vasculogenic mimicry and its association with prognosis in cancer.

**Number of patients (percentage VM+)**	**Association**	***p*-value**	**References**
331 (7.9%)	VM group tended to have a lower 60-month survival rate than the non-VM group	*p* = 0.071	(18)
	VM group tended to have a higher hematogenous metastases than the non-VM group	*p* = 0.059	
90 (28.6%)	VM correlated with lymph node metastases	*p* = 0.004	(26)
	Histological grade	*p* < 0.001	
	Nottingham prognostic index (NPI) (worse prognosis)	*p* < 0.001	
	No correlated with the presence of:		
	ER	*p* = 0.391	
	PR	*p* = 0.321	
	Her2	*p* = 0.114	
	VM correlated with overall survival	*p* < 0.001	
	And disease-free survival	*p* < 0.001	
200 (30%)	VM and Osteopontin co-expression correlated with pathological complete response	*p* = 0.006	(27)
202 (16.8%)	VM presence was higher in TNBC vs. non TNBC	*p* = 0.003	(28)
	VM correlated with worst	*p* < 0.001	
	Disease free survival and overall survival	*p* = 0.015	
134 (30.6%)	VM presence was higher in TNBC vs. non TNBC	*p* = 0.004	(29)
100 (29%)	VM presence was higher in TNBC vs. non TNBC	*p* = 0.020	(30)
	VM correlated with poorer overall survival	*p* = 0.015	
174 (24.7%)	VM presence was higher in TNBC vs. non TNBC	*p* = 0.044	(31)
120 (22.5%)	VM correlated with positive node status;	*p* = 0.027	(32)
	a higher tumor stage	*p* = 0.022	
	and higher levels of HER2	*p* = 0.018	
	VM did not correlate with ERalpha or PR status	*p* = 0.143	

## The Role of CSCS in VM

In normal adult tissues, there are cells with the ability to proliferate, self-renew, and differentiate that allow tissue regeneration. These cells are known as stem cells. Similarly, it has been proposed that in malignant tumors, there is a cell subpopulation with the ability to self-renew and undergo less differentiation. In addition, it is hypothesized that these cells show mesenchymatous features, higher invasive capacity, and improved resistance to chemotherapeutic treatment. These cells have been called Cancer Stem Cells (CSCs). CSCs are characterized by specific markers, including CD44, CD133, CD166, ABC transporters, or metabolic enzymes such as Aldehyde dehydrogenase-1 (ALDH1) (45). It has recently been described that in different types of cancer, cells with stem characteristics actively participate in the formation of VM (46).

In human breast tumor xenografts transplanted in mice, it was demonstrated that a CD44+CD24– cell subpopulation presented CSC characteristics. CD44+CD24– cells obtained from mouse tumors were able to form tumors in other mice when as few as 1,000 cells were injected, while CD44+CD24+ cells did not form tumors even when injected more than 10,000 cells. In addition, tumors formed from CD44+CD24– cells presented cell heterogeneity, demonstrating that these cells were able to differentiate into a heterogeneous tumor (47). On the other hand, ALDH1 expression has been shown to be a marker of stem cells in normal tissue and breast tumors. In murine models, ALDH1+ cells derived from breast tumors were shown to have a superior ability to form tumors. Furthermore, the expression of ALDH1 is associated with lower overall survival and a higher probability of developing metastases in breast cancer patients (48, 49). In addition, the presence of ALDH1 is associated with the formation of VM. Both factors were shown to be associated with poorer overall and disease-free survival. Both the expression of ALDH1 and the presence of VM were most prevalent in triple-negative tumors (28). In an *in vitro* model using the HCC1937/p53 cell line (a triple-negative cell line with inducible p53 transfection) it was observed that ALDH1A3+ cells (one of the isoforms of the ALDH1 enzyme) could form tubular structures when they were grown in matrigel, while ALDH1A3- cells were not capable of creating such structures. The expression of ALDH1A3 coincided with the presence of Ki67, a proliferation marker, so it is inferred that cells that express the stem cell marker also have a greater proliferative capacity (50). On the other hand, in a study that included 134 samples of breast cancer patients, it was demonstrated that the CD133 marker was associated with VM in different breast cancer subtypes. The subtype that presented a more significant number of cases with VM and vessels with higher volume was the triple-negative. In addition, in the MDA-MB-231 cell line, a subpopulation characterized by the expression of the CD133 marker was described. This subpopulation was able to establish vessels in a matrix and expressed VE-cadherin and the metalloproteinases MMP-2 and MMP-9 (29). Therefore, as in other tumors, CSCs in breast tumors are actively involved in the formation of vessels of tumor origin. However, not all reports agree on the specific presence of CSC markers and the presence of VM. For example, Sun et al. found an association between VM formation and the presence of ALDH1 and CD44+CD24– phenotype, but not with the presence of CD133 (30). Therefore, it will be important to determine whether there is a single type of CSC in breast cancer or whether populations with stem cell characteristics are variable among tumors. Ginestier et al. demonstrated that only a fraction of the ALDH1-positive cells also possesses the CD44+CD24– phenotype. In addition, these cells had greater tumorigenic capacity compared to those with only one or none of these markers (49). Hence, stem cell markers used so far in breast cancer are not universal and may represent variants, sometimes synergistic, but with specific characteristics relevant to the treatment and progression of breast cancer.

## VM in Triple-Negative Tumors

Triple-negative Breast Cancer (TNBC) includes a heterogeneous group of tumors characterized by the absence of expression of ER, PR, and that do not possess overexpression or HER2 amplification. Although these tumors have a high response to chemotherapy, they also have a poor prognosis for overall survival and relapse (51, 52). There is a higher proportion of triple-negative tumors with VM compared to tumors positive for ER, PR, and/or HER2. Accordingly, these tumors also have a greater number of vessels formed by VM (28–31). However, this association is controverted (26, 32). Indeed, Liu et al. found a correlation between the expression of HER2 and VM (32). Nonetheless, none of these studies grouped triple negative tumors, and the ER and PR, or HER2 mark were evaluated independently. Finally, *in vitro* analyses have shown differences in the ability to form vessels by VM and the mechanisms involved in this process between TNBC and no-TNBC cells. However, most of these studies use the MDA-MB-231 cell line as the TNBC tumor model and, only occasionally compare it to a different cell line. Despite the importance of the MDA-MB-231 line as a breast cancer study model, it is difficult to make a generalization regarding all TNBC tumors, due to their heterogeneity between patients and even within single tumors (40, 42, 44, 53). Although vessel formation by VM is more common in TNBC tumors, it is not exclusive to this type of tumor. However, due to the lack of specific therapies in this group, VM inhibition is a good candidate for therapeutic targeting.

## Relationship Between CSCS, VM and the Epithelial-Mesenchymal Transition

As mentioned above, the presence of CSC markers is associated with the formation of VM. In addition, other factors related to morphological and cellular motility have a role in VM. During tumor progression, the epithelial-mesenchymal transition refers to the change of epithelial tissue, with very close cells interacting through intercellular unions to a mesenchymal-like tissue, i.e., cells with greater invasive capacity, a large amount of intercellular material and without the apicobasal polarity characteristic of the epithelium. (54, 55). The epithelial-mesenchymal transition is regulated by three families of transcription factors: SNAI (SNAI1/Snail and SNAI2/Slug), ZEB (Zinc finger E-box-binding homeobox; ZEB1 and ZEB2) and TWIST (TWIST1 and TWIST2). The activation of these gene families has been described. Nonetheless, the main mechanism by which these transcription factors promote TMS is through the repression of genes essential for the epithelial structure, such as *CDH1*, which encodes for E-cadherin, involved in adherens junctions (56). These factors bind epigenetic regulators and, together, regulate gene expression. For example, TWIST1 increases the expression of *BMI1* (a repressor complex of the Polycomb family), and both are essential to repress the expression of *CDH1* (57, 58). TGF-beta is one of the pathways that initiate the epithelial-mesenchymal transition. TGF-beta is a family of ligands that bind serine/threonine kinase receptors. In turn, these receptors phosphorylate and activate SMAD proteins. Finally, SMAD activation regulates the transcription of factors associated with EMT, such as SNAI1 or ZEB. On the other hand, it has been observed that the activation of the EMT program entails the cellular acquisition of CSC characteristics (Figure 2B) (54). In immortalized cells of breast epithelium, the overexpression of SNAIL increases the percentage of CD44+CD24– cells. Furthermore, CD44+CD24– cells show EMT-distinctive morphology. This phenomenon was also observed in cells transformed by the introduction of the *HER2/neu* oncogene (59). Therefore, EMT promotes the occurrence of CSCs in breast cancer.

Both EMT and CSC are related to VM. In TA2 mice (a mammary tumor model) MDA-MB-231 xenograft tumors, hypoxia-induced with the anti-angiogenic agent sunitinib is associated with VM and an increase in CD133+ cells. In addition, in matrigel cell cultures, activation of the HIF1alpha factor promotes *TWIST1* transcription, which increases the percentage of CD133+ cells and vessel formation through VM. In this model, the inhibition of *TWIST1* prevents the formation of VM and the emergence of cells with stem markers (31). On the other hand, the ZEB1 factor decrease in MDA-MB-231 cells inhibits VM and increases the expression of E-cadherin. In doing so, EMT is reversed while VM is inhibited (60). In breast cancer tumors, overexpression of TWIST is associated with a lower expression of epithelial factors such as E-cadherin (61, 62). Increased TWIST levels also correlate with more advanced stage tumor and are more common in TNBC tumors and HER2+ (63). Overexpression of TWIST and SLUG has also been observed in stromal tumor cells (64). However, the association of TWIST expression with disease-free survival and overall survival has not been consistently observed throughout these studies (63–65). Moreover, in a sample of 100 breast tumors, Nodal expression was associated with VM formation and VE-cadherin expression in a subgroup of tumors. Nodal is part of the TGF-beta family and participates in the development and regulation of differentiation (66). *In vitro* studies have demonstrated that Nodal expression is necessary for the formation of vessels by VM (67). Therefore, EMT, VM, and the presence of CSCs are interrelated and not isolated phenomena. Common features are the change toward an epithelium with invasiveness and migration capacity, less differentiation, and the ability to create tumor vessels.

## VM as a Therapeutic Target

As mentioned above, VM vessel formation is a process that includes proliferation, migration, invasiveness, and alterations in intercellular junctions. Accordingly, therapeutic inhibition of VM can target any of these processes. For example, it has been proposed that the use of a cytotoxic drug such as vincristine in combination with a specific inhibitor of the sarcoma family kinases (SFKs), which regulate signaling pathways involved in processes associated with VM, could have an additive effect on VM inhibition. In fact, an *in vitro* model using liposomes showed that both drugs can cause cell death and inhibition of vessel formation in MDA-MB-231 cells grown in matrigel. In addition, the use of these liposomes decreases the tumor volume of xenografts in nude mice (68). The authors also demonstrated that the use of liposomes for transporting compounds with different targets, such as epirubicin (a DNA intercalant) and celecoxib (a cyclooxygenase 2 inhibitor) are able to inhibit VM in breast cancer cells (69). On the other hand, it has also been proposed that the best strategy to inhibit vessel formation in tumors will be the simultaneous inhibition of angiogenesis and VM. New drugs, like acridine in complex with metals, such as gold, have shown the ability to promote apoptosis of cancer cells and inhibit the formation of vessels formed by endothelial cells (angiogenesis) or cancer cells (VM) (70).

The use of compounds obtained from natural extracts, such as brucine, has also been associated with VM inhibition. Brucine inhibits migration and invasiveness of MDA-MB-231 cells (71). Besides, brucine modifies the structure of actin and tubulin cytoskeleton and inhibits the formation of vessels by VM (72). Hinokitiol is also a natural compound with anti-tumor properties. In cells obtained from mammospheres, it was demonstrated that hinokitiol diminishes levels of the EGFR protein by increasing its proteasome-mediated degradation and, consequently, inhibits VM (73).

On the other hand, vessel formation by VM depends on the EGFR receptor in CSCs ALDH+ derived from breast tumors (74). Another compound that has demonstrated the ability to inhibit vessel formation by VM is 6'-bis (2.3-dimethoxybenzoyl)-a,a-D-trehalose (DMBT) a derivative of brartemicin, a metabolite isolated from actinomycetes (75).

Currently, there are no specific therapies to inhibit VM. However, it is possible to propose that epithelial-mesenchymal transition, invasiveness and the presence of cancer stem cells may be useful targets to slow the formation of vessels by VM, in addition to having antitumor effect *per se*.

## Conclusion

VM is an alternative mechanism to angiogenesis that allows vessel formation without the involvement of endothelial cells. These vessels provide nutrients to the tumor and can serve as a means of spreading cancer cells. The absence of a therapeutic benefit of anti-angiogenic therapies in breast cancer may be due to the formation of vessels by VM. In addition, the formation of vessels with tumor cells may be a factor explaining the increased aggressiveness of tumor subtypes such as TNBC. However, VM also occurs in other breast cancer subtypes. The role of CSCs in VM in breast cancer will be better defined when specific stem markers are found to classify these cells. In addition, it will be important to use a greater variety of *in vitro* and *in vivo* models of breast cancer cells to determine the specific factors associated with the formation of VM in breast cancer. Finally, the discovery of particular factors involved in VM in breast cancer will make it possible to more precisely target therapies that inhibit the formation of vessels and may affect several processes that are important for tumor progression.

## Author Contributions

JD-C conceived and designed the structure of the review and revised the manuscript. MA-E, YA-M, RC-G, and LH contributed in the manuscript writing and CC-S performed the CD31-PAS double staining and histopathology description. All authors have read and approved the final version of the manuscript.

### Conflict of Interest

The authors declare that the research was conducted in the absence of any commercial or financial relationships that could be construed as a potential conflict of interest.
